# Umbilical cord bilirubin as a predictor of neonatal jaundice: a retrospective cohort study

**DOI:** 10.1186/s12887-017-0938-1

**Published:** 2017-09-20

**Authors:** Kelsey D. J. Jones, S E. Grossman, Dharshini Kumaranayakam, Arati Rao, Greg Fegan, Narendra Aladangady

**Affiliations:** 1 Neonatal Unit, Homerton University Hospital NHS Foundation Trust, London, UK; 2 Department of Paediatrics, Imperial College, London, UK; 3 Barts and the London School of Medicine & Dentistry, Queen Mary, University of London, London, UK; 4 Swansea Trials Unit, School of Medicine, Swansea University, Swansea, UK

**Keywords:** Neonatology, Jaundice, Haematology

## Abstract

**Background:**

Hyperbilirubinaemia is a major cause of neonatal morbidity. Early identification of those infants most at risk might allow the development of targeted primary preventative therapy and follow-up. The objective of this study was to assess whether arterial umbilical cord bilirubin (aUCB) level at delivery predicts the development of neonatal jaundice in term deliveries.

**Methods:**

Retrospective analysis of hospital biochemistry records identified term deliveries with recorded aUCB. Infant medical records were reviewed to identify those who developed neonatal hyperbilirubinaemia (requiring treatment according to UK NICE guidelines) with/without a positive direct antiglobulin test (DAT).

**Results:**

Of 1411 term deliveries with a clearly recorded aUCB, 30 infants developed clinically-significant jaundice (2.7%), of whom 8 were DAT + ve (0.6%) mostly due to ABO incompatibility. aUCB strongly predicted the development of DAT + ve jaundice (area under the ROC curve = 0.996), as well as all-cause jaundice (area under the ROC curve = 0.74). However, this effect was critically dependent on maternal blood group. Amongst infants at risk of ABO incompatibility (maternal blood groups O + ve/O-ve, 39.7%) the predictive value of aUCB for all cause jaundice was strengthened (area under the ROC curve = 0.88). Amongst those not at risk (defined maternal blood group not O + ve/O-ve, 51.0%) it disappeared completely (area under the ROC curve = 0.46). A cutoff of 35 μmol/l for mothers with blood group O + ve/O-ve increased the pre-test probability for all-cause jaundice of 4% to a post-test probability of 30%.

**Conclusions:**

For infants of mothers with blood group O, aUCB predicts development of neonatal jaundice. There was no evident utility for infants of mothers with other blood groups. Estimation of aUCB should be considered as a strategy for early identification of those at risk of neonatal haemolytic jaundice.

## Background

Hyperbilirubinaemia is one of the commonest causes of admission to hospital in the neonatal period amongst term babies in all settings [1–3]. Prevention of serious complications depends on effective early treatment, but clinically significant jaundice may not develop until one or more days after delivery. Current practice, which usually promotes early discharge after delivery, may introduce delays in recognition and initiation of medical therapy [4, 5]. Identification of biomarkers that could be measured within a few hours following birth, which robustly predict incident jaundice, would represent a significant advance.

Estimation of umbilical cord blood bilirubin (UCB) at delivery is practicable, cheap and non-invasive. It could be easily integrated with the current trend towards routine umbilical cord blood biochemical evaluation practiced in many centres. Several previous studies have investigated the potential utility of UCB estimation in predicting subsequent hyperbilirubinaemia [6–12]. Results have been inconsistent, and a review by the UK National Institute for Health and Care Excellence (NICE) concluded that it was not a useful index of risk [13].

In this study we exploited the fact that at our institution UCB estimation is routine, and performed a retrospective analysis to assess the potential utility of arterial umbilical cord bilirubin (aUCB) in predicting clinically significant hyperbilirubinaemia, and hyperbilirubinaemia due to neonatal haemolytic disease.

## Methods

Homerton University Hospital is an inner-London District General Hospital providing maternity care to one of the most economically deprived areas in the UK. The local population is ethnically diverse, with large Afro-Caribbean, Turkish, and Orthodox Jewish communities. The obstetric unit is a local referral centre with approximately 5000 deliveries annually, 9% of which are below 2.5 kg. The delivery unit combines midwife and obstetric-led care, and is equipped with a GEM4000 whole blood analyzer, which is operated by delivery suite staff and used for umbilical cord gas analysis at delivery. For this project we have exploited the fact that it is departmental policy to perform umbilical cord gas analysis on all obstetric-led deliveries, and that the GEM4000 automatically provides total bilirubin estimation on such samples.

From an electronic database backup of the delivery unit whole blood analyzer, we extracted data on all samples processed in the 9 months from February to November 2010. We used the hospital case number and date of birth to identify and group all samples belonging to a single individual (mother), and used these data to interrogate the electronic patient records of each mother and infant pair.

Because it is well recognised that paired umbilical cord blood samples are frequently mislabelled, we only considered samples to be a true arterial cord blood sample if there was another sample with the same hospital case number processed within 30 min, with a pH that was higher by >0.02. This accounts for the fact that umbilical cord veins are easier to sample than arteries such that where a single or two identical samples are received they are most likely to be venous regardless of how they have been labelled [14].

We recorded clinical and demographic factors including maternal blood group (in the UK all pregnant women are offered prenatal blood group analysis to assess for risk of Rhesus-incompatibility disease), gestation at delivery (estimated by dating ultrasound scan or last menstrual period), the infant’s sex and birthweight. We recorded maternal ethnicity because of ethnically-defined differences in some causes of neonatal jaundice (e.g. glucose-6-phosphate dehydrogenase deficiency (G6PD)). Electronic notes and blood test results from the delivery admission and any subsequent readmissions were scanned by two experienced paediatricians (KJ and AR, who were blinded to the aUBC level) in order to identify all infants who developed clinically significant jaundice. This was defined as a single total bilirubin result above the phototherapy treatment threshold on charts from the UK’s NICE Guideline on Neonatal Jaundice [13]. We scanned electronic neonatal notes and cross-checked with separate laboratory databases to record positive direct antiglobulin tests (DAT) and low glucose-6-phosphate dehydrogenase levels for each term infant with an arterial cord blood sample.

Single variable logistic regression (for continuous or ordinal variables) or chi-squared (for categorical variables) analyses were performed to screen for association with clinically significant neonatal jaundice, and with DAT-positive (haemolytic) neonatal jaundice. Variables demonstrating significant associations (*p* < 0.05) were included in a multivariable analysis. Receiver operator characteristic (ROC) curves were plotted. Subgroup analyses was performed amongst those mothers with either O-ve or O + ve blood groups (whose infants are at risk of ABO incompatibility haemolytic disease), and mothers with a defined blood group that was neither O-ve or O + ve (whose infants are not at risk of ABO incompatibility). In our setting ABO incompatibility is the commonest cause of haemolytic jaundice. Analysis was performed in Stata version 11.0.

The study was reviewed and approved by the UK National Research Ethics Service (NRES) Committee London – Harrow (reference 11/LO/0796) and received institutional approval from Homerton University Hospitals Research and Development Department.

## Results

Over the 9-month period, there were 4069 inborn deliveries, on whom umbilical cord blood analysis had been performed on 2128 (52.2%). Of these, 263 (12.3%) were premature (gestation less than 37 weeks) and 432 (20.3%) did not have an umbilical cord blood result that could clearly be identified as arterial. Medical notes were missing in 22, which left 1411 (66.3%) mother-infant pairs who were included in the analysis.

3.7% of mothers had O-ve blood group, and 36.0% were O + ve (39.7% group O, overall). In terms of self-defined ethnicity, 27.3% of mothers were black (African or Caribbean origin), 7.3% were south Asian, and 6.3% were Middle Eastern. Median birthweight was 3390 g (interquartile range (IQR) 3080 to 3680 g), and median gestation in weeks + days was 40 + 0 (IQR 39 + 0 to 41 + 0, range 37 + 0 to 43 + 0). 52.4% of deliveries were male.

There were 30 episodes of clinically significant jaundice (2.1%). 9 infants were found to be DAT + ve (0.6%) of whom 8 were jaundiced, and there was a single case of G6PD deficiency. One case of DAT + ve jaundice was due to Rhesus incompatibility (O-ve mother), and the rest were due to ABO incompatibility (O + ve mothers).

**Fig. 1:**
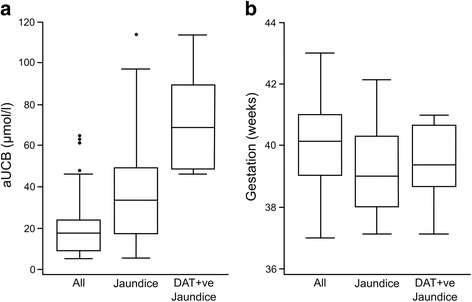
**a** shows aUCB levels amongst all infants, infants with all-cause jaundice (*P* < 0.001), and infants with DAT + ve jaundice (P < 0.001). **b** shows gestation amongst all infants, infants with all-cause jaundice (*P* = 0.02), and infants with DAT + ve jaundice (*P* = 0.28)

Gestation and aUCB were both significantly associated with development of clinically significant jaundice (*P* = 0.001 and *P* < 0.001, respectively). Maternal blood group was only significant when dichotomised into those potentially at risk of ABO incompatibility (blood groups O + ve or O-ve) or not (other blood groups) (*P* = 0.02). Apart from blood group, the only variable associated with DAT + ve jaundice was aUCB (P < 0.001), as shown in Fig. 1.

**Fig. 2:**
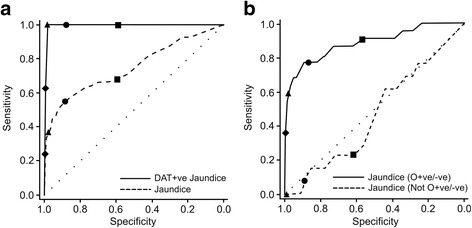
**a** shows ROC curves for aUCB predicting all cause jaundice (dashed line) or DAT + ve jaundice (thick line) amongst all infants. **b** shows ROC curves for all-cause jaundice amongst infants at risk of ABO incompatibility (i.e. with maternal blood group O + ve or O-ve, thick line), and amongst those infants not at risk (i.e. documented maternal blood group not O + ve or O-ve, dashed line). In both instances the symbols denote aUCB cutoffs (in μmol/l) as follows: Diamond = 50, Triangle = 40, Circle = 30, Square = 20

ROC curves demonstrate that amongst all infants, aUCB strongly predicts the development of DAT + ve jaundice (area under the ROC curve = 0.996 (95% CI 0.991 to 0.998); Fig. 2a), as well as all-cause jaundice (area under the ROC curve = 0.75 (95% CI 0.72 to 0.77); Fig. 2a) even when DAT + ve cases are omitted (area under ROC curve = 0.68 (95% CI 0.66 to 0.71); graph not shown).

We separated out infants at risk of ABO incompatibility (i.e. whose mothers were blood groups O + ve or O-ve) from those certainly not (with a recorded blood type not O + ve or O-ve) and considered the groups separately. Amongst 560 (39.7%) infants at risk of ABO incompatibility, aUCB had similar specificity but substantially higher sensitivity for predicting all-cause jaundice than when all infants were considered, with an area under the ROC curve of 0.89 (95% CI 0.86 to 0.91). The area under the ROC curve for DAT + ve jaundice in this group was 0.994 (0.982 to 0.998). Interestingly, the predictive effect was completely lost for those not at risk of ABO incompatibility (720 infants, 51.0%), with an area under the ROC curve of 0.46 (95% CI 0.43 to 0.50; Fig. 2b). Furthermore, considering only those at risk of ABO incompatibility the effect of gestation on risk of all-cause jaundice was no longer significant (*P* = 0.06).

The aUCB cutoff levels and sensitivity and specificity for predicting all-cause and DAT + ve jaundice are presented in Tables 1 and 2. Furthermore, since the rate of clinically significant jaundice, at 2.1% (3.9% in O + ve/−ve mothers), was similar to that found in other studies in similar settings, we also calculated positive and negative predictive values at the different cutoffs [15–17].

**Table 1:** aUCB and all-cause jaundice

aUCB (μmol/l)	All subjects						Only O + ve/O-ve Mothers					
	Sens (%)	Spec (%)	PPV (%)	NPV (%)	LR+	LR-	Sens (%)	Spec (%)	PPV (%)	NPV (%)	LR+	LR-
>20	68.4	59.2	4.4	98.6	1.7	0.53	90.9	56.1	7.8	99.3	2.1	0.16
>25	63.2	76.0	6.8	98.7	2.6	0.48	86.4	73.2	11.7	99.2	3.2	0.19
>30	55.3	88.2	11.5	98.6	4.7	0.51	77.3	86.4	18.9	98.9	5.7	0.26
>35	44.7	94.2	17.7	98.4	7.7	0.59	68.2	93.9	31.3	98.6	11.1	0.34
>40	36.8	97.7	30.4	98.2	15.8	0.65	59.1	97.4	48.1	98.3	22.7	0.42
>45	31.6	98.8	42.9	98.1	27.1	0.69	50.0	98.7	61.1	98.0	38.4	0.51
>50	23.7	99.5	56.3	97.9	46.5	0.77	36.4	99.3	66.7	97.4	48.9	0.64

*Sens* sensitivity, *spec* specificity, *PPV* positive predictive value, *NPV* negative predictive value, *LR+* positive likelihood ratio, *LR-* negative likelihood ratio

**Table 2:** aUCB and DAT + ve jaundice

aUCB (μmol/l)	All subjects						Only O + ve/O-ve Mothers					
	Sens (%)	Spec (%)	PPV (%)	NPV (%)	LR+	LR-	Sens (%)	Spec (%)	PPV (%)	NPV (%)	LR+	LR-
>20	100	58.8	1.4	100	2.4	0.00	100	55.1	3.1	100	2.2	0.00
>25	100	75.4	2.3	100	4.1	0.00	100	71.9	4.9	100	3.6	0.00
>30	100	87.5	4.4	100	8.0	0.00	100	85.1	8.9	100	6.7	0.00
>35	100	93.7	8.2	100	15.8	0.00	100	92.8	16.7	100	13.8	0.00
>40	100	97.3	17.4	100	36.9	0.00	100	96.6	29.6	100	29.1	0.00
>45	100	98.6	28.6	100	70.2	0.00	100	98.2	44.4	100	55.2	0.00
>50	62.5	99.2	31.3	99.8	79.7	0.38	62.5	98.7	41.7	99.5	49.3	0.30

*Sens* sensitivity, *spec* specificity, *PPV* positive predictive value, *NPV* negative predictive value, *LR+* positive likelihood ratio, *LR-* negative likelihood ratio

Finally, since all subjects in the study had verifiable arterial and venous samples, we assessed the correlation between arterial and venous bilurubin levels. The correlation coefficient was 0.80 (*P* < 0.001).

## Discussion

This study demonstrates that aUCB is potentially useful screening tool for haemolytic jaundice in term infants of mothers with blood groups O + ve or O-ve. Although the study design has several limitations, it is the largest study to date investigating the role of umbilical cord bilirubin in predicting neonatal jaundice in term babies, and identifies a subgroup of infants in whom aUCB screening may have considerable utility. Infants with high aUCB could be subjected to primary preventative measures such as early feeding, prophylactic phototherapy, and enhanced monitoring, with the aim of preventing severe jaundice.

Consideration of umbilical cord bilirubin as an index of risk for neonatal jaundice is not a new idea. It has been an area of interest since the 1950s, and most studies have found it to be a useful predictor to a greater or lesser extent [6]. Comparison and meta-analysis of studies has been complicated by the fact the most have tended to consider aUCB as a categorical instead of continuous variable. Substantial differences in reported sensitivity and specificity values may simply have reflected the use of different arbitrary cutoffs. For example, Knupfer et al. found that using a cutoff of 30μmol/l to predict neonatal hyperbilirubinaemia had very high sensitivity and low specificity of 97% and 41.4% respectively (compared to 88.2% and 55.3% for that cutoff in our study), whereas Carbonell et al. found that an aUCB cutoff of 37μmol/l had low sensitivity but high specificity of 22.2% and of 94.7%, (compared to 44.7% and 95.6% in our study) [8, 10]. Both studies have results not dissimilar to those found in our analysis, even though their reported conclusions appear to be contradictory. In addition, direct comparison between studies may be misleading as most do not make clear whether arterial, venous or a mixture of umbilical samples were analysed. Our data suggest that venous and arterial values are strongly correlated, but that venous levels are consistently lower than arterial, leaving open the possibility of systematic error when comparing between study groups.

A significant advantage of the current study is that infants at risk of the most common cause of haemolytic disease of the newborn in our setting (ABO incompatibility) have been considered separately to those not at risk, and that the number of events in both arms is sufficient to meaningfully compare the two. The data strongly suggest that aUCB should be considered a predictor of jaundice due to haemolytic disease rather than all-cause jaundice, and that umbilical cord bilirubin estimation has no predictive value for jaundice in infants not at risk of haemolysis. This presents a further reason why comparison between studies is problematic, because it suggests the predictive characteristics of aUCB will be partly determined by percentage of the population at risk of haemolytic causes of jaundice, which is subject to geographic variation. Although these results mainly reflect jaundice due to ABO incompatibility, the two infants with haemolysis not caused by ABO also had very high aUCB; the only infant with proven Rhesus-incompatibility disease had the highest aUCB recorded in the study period at 113.9μmol/l, and the infant with G6PD manifesting as early jaundice had an aUCB of 51μmol/l, on the 99th centile of our data.

The study has a number of limitations. Umbilical cord blood specimens were not systematically processed during the study period, with analysis undertaken in only 52% of inborn deliveries. Cord blood analysis was not generally undertaken in deliveries under midwifery-led care, introducing an important bias into our dataset towards complicated pregnancies and deliveries requiring medical intervention. Although these features might influence the probability of developing neonatal jaundice, it was reassuring to see that the jaundice rate in our sample was in line with previous population-based studies. Furthermore the retrospective data capture is incomplete with substantial gaps, for example maternal blood group is missing in 3.3% of cases. The study presents data from a single centre that is atypical compared to the wider UK population, serving an economically deprived and unusually ethnically diverse catchment area. Although our efforts to identify infants with each of the three outcomes have been thorough, it is difficult to exclude that some who did develop jaundice have been miscategorised as not developing it, which could lead to overestimation of sensitivity. It is disappointing that it was not possible to identify an unambiguous arterial sample in such a high proportion of cases. It may be that future studies focus on venous levels, although occasional inadvertent sampling of the artery will compromise specificity in that case.

In our dataset small changes in aUCB cutoffs may be associated with substantial alterations in sensitivity and specificity for predicting hyperbilirubinaemia. The exact level of an ‘ideal’ cutoff, will probably be different between different centres serving distinct populations. In our setting, a cutoff of 35 μmol/l for mothers with blood group O + ve or O-ve had a positive predictive value of almost a third for predicting early neonatal jaundice, increasing the pre-test probability of 4% to a post-test probability of 30%.

## Conclusion

This study proves the concept that umbilical cord bilirubin is predictive of the development of neonatal jaundice, especially haemolytic jaundice, in infants of O + ve and O-ve mothers. It should prompt re-consideration of the applicability of this practical, cheap and non-invasive approach.

## Abbreviations

aUCB: Arterial umbilical cord bilirubin
DAT: Direct Antiglobulin Test
G6PD: Glucose-6-phosphate dehydrogenase deficiency
IQR: Interquartile range
NICE: UK National Institute for Health and Care Excellence
ROC: Receiver operator characteristic
UCB: Umbilical cord bilirubin
UK: United Kingdom
